# Current and emerging adjuvant therapies in biliary atresia

**DOI:** 10.3389/fped.2022.1007813

**Published:** 2022-10-14

**Authors:** Scott C. Fligor, Thomas I. Hirsch, Savas T. Tsikis, Andrew Adeola, Mark Puder

**Affiliations:** Vascular Biology Program, Department of Surgery, Boston Children’s Hospital, Harvard Medical School, Boston, MA, United States

**Keywords:** biliary atresia, therapeutics, cholestasis, cholangitis, liver transplant, glucocorticoids, survival, children

## Abstract

Following Kasai hepatic portoenterostomy (HPE), most patients with biliary atresia will eventually require liver transplantation due to progressive cirrhosis and liver failure. Preventing liver transplantation, or even delaying eventual liver transplantation, is the key to improving long-term outcomes. This review first examines the commonly used adjuvant therapies in post-HPE biliary atresia and the strength of the evidence supporting these therapies. Next, it examines the evolving frontiers of management through a comprehensive evaluation of both recently completed and ongoing clinical trials in biliary atresia. Promising therapies used in other cholestatic liver diseases with potential benefit in biliary atresia are discussed. Improving post-HPE management is critical to prevent complications, delay liver transplantation, and ultimately improve the long-term survival of patients with biliary atresia.

## Introduction

Biliary atresia is a fibro-inflammatory disease affecting both intra- and extrahepatic bile ducts and presenting with biliary obstruction (1, 2). Although rare, affecting one in 10,000–20,000 live births, biliary atresia is the most common indication for liver transplantation in childhood (2–5). The Kasai hepatic portoenterostomy (HPE) is the primary palliative procedure for restoring bile flow (6). The obliterated extrahepatic bile ducts are transected with anastomosis between the small intestine and the dissected porta hepatis to allow bile drainage via microscopic bile ducts. In modern series, half of patients survive a decade with their native liver and over 90% survive into adulthood (7, 8). However, most patients ultimately require liver transplantation as the definitive treatment for progressive native liver disease following HPE. In patients with late diagnosis and advanced liver disease at time of diagnosis, primary liver transplantation may be required.

Long-term survival depends upon delaying eventual transplantation. Patients under two years of age have higher waitlist and posttransplant mortality (9). The most important prognostic factor following HPE is successful bile drainage characterized by clearance of jaundice. Infants with successful clearance of jaundice versus those without demonstrate remarkably higher survival with their native liver at two years (86% vs. 20%) (10). Earlier diagnosis and HPE, particularly within the first 30–45 days of life, results in higher successful clearance of jaundice and improved native liver survival (11). Stool color cards are the most commonly instituted neonatal screening to facilitate early diagnosis and expeditious HPE (12).

Native liver disease in post-HPE patients includes recurrent cholangitis, cholestasis, synthetic liver failure, cirrhosis, portal hypertension, and malignancy (13, 14). Initially, native liver disease presents as a chronic cholangiopathy with bile ductular proliferation and liver fibrosis (15, 16). Molecular profiling of tissue samples obtained at HPE demonstrate distinct histologic phenotypes: inflammation-predominant, mixed, or fibrosis-predominant (17). The inflammation-predominant phenotype was found in younger patients at time of HPE and was associated with better outcomes, suggesting a pathologic progression from initial inflammation to fibrosis with implications for long-term survival of the native liver (17). While a massive inflammatory response appears to be a key part of the initial pathogenesis of biliary atresia, post-HPE inflammation may be pro-reparative (18).

The initial goals following HPE are successful bile drainage, prevention of progression of native liver disease to cirrhosis, prevention of cholangitis (which accelerates the progression to cirrhosis), and achievement of satisfactory growth and development. Thus, interventions in post-HPE patients generally focus on choleresis, preventing inflammation, preventing cholangitis, and ensuring adequate nutrition.

Few medical therapies have been rigorously investigated in biliary atresia and found to substantially alter the progression to transplantation. Here, we review the evidence for the current management of post-HPE biliary atresia and discuss emerging investigational therapies.

## Current adjuvant therapies for biliary atresia

### Glucocorticoids

Glucocorticoids are among the most studied interventions following HPE for biliary atresia (19–27). The concept of “blast therapy” originated with Karrer and Lilly in 1975: a four day taper of high dose methylprednisolone (starting dose 10 mg/kg) increased bile flow and decreased serum bilirubin in post-HPE patients with cholangitis or decreased bile flow (28). Glucocorticoids also have profound anti-inflammatory effects (29). Current evidence for glucocorticoids in post-HPE biliary atresia is weak, although the data suggest a possible benefit especially in clearance of jaundice.

The highest quality evidence consists of three randomized placebo-controlled trials (19, 21, 26). Davenport et al. conducted a randomized, double-blind, placebo-controlled trial at two centers in the United Kingdom (26). In the prednisolone group, 26% underwent transplant at 12 months compared to 35% in the placebo group (*P = *0.47), while 50% had normal bilirubin in the prednisolone group compared to 40% in the placebo group (*P *= 0.35). A subgroup analysis of infants undergoing early portoenterostomy (age < 70 days) found decreased median bilirubin at one month (64 vs. 116 umol/L, *P = *0.01), but this was not sustained at six months.

The START trial was a multi-center, double-blind, randomized, placebo-controlled trial conducted at 14 United States centers (21). Infants were randomized to a 13-week steroid course or placebo (*n* = 70). There was no significant difference between the steroid group and control group in the primary endpoint of successful bile drainage at six months post-HPE in either the intention-to-treat (58.6% vs. 48.6%) or per-protocol analysis. In infants <70 days old at time of HPE, there was similarly no significant difference between the steroid and control groups for successful bile drainage (71.8% vs. 56.8%). There was similar survival with native liver at 24 months post-HPE (58.7% vs. 59.4%, *P *= 0.99) and successful bile drainage at 24 months (49.4% vs. 39.8%, *P *= 0.29).

A recent randomized controlled trial at a single center in China randomized infants post-HPE (performed by a single surgeon) to steroid treatment or placebo (19). The primary outcome of clearance of jaundice with native liver at six months was significantly higher in the steroid group compared to the placebo group (54.1% vs. 31.0%, *P *= 0.0015) with higher native liver survival rate at 24 months (57.1% vs. 40.0%, *P *= 0.03). While this study is limited in generalizability due to its single center and single surgeon experience, these data are consistent in direction and magnitude with the previously described randomized controlled trials and provide weak support for post-HPE glucocorticoids in biliary atresia.

### Antibiotic prophylaxis

Cholangitis affects 40%–93% of post-HPE patients, most commonly in the first two years of life (30). Prophylactic antibiotics are often prescribed to prevent cholangitis and, hopefully, prevent the progression of liver disease. Surveys of primarily European and North American centers have found most utilize prophylactic antibiotics post-HPE, although there is minimal evidence to support a specific antibiotic regimen or duration of prophylaxis (30, 31).

There are no randomized placebo-controlled trials evaluating antibiotic prophylaxis. A small single center trial in Taiwan randomized patients with one episode of cholangitis post-HPE to prophylaxis with trimethoprim-sulfamethoxazole (TMP/SMZ; *n* = 9) or neomycin (*n* = 10), compared to a historical control with no prophylactic antibiotics (*n* = 18) (32). Patients in both antibiotic groups had decreased cholangitis recurrence compared to the historical control group (TMP/SMZ: RR 0.52 [95% CI 0.29, 0.98], neomycin: RR 0.42 [95% CI 0.22,0.82]). A recent randomized controlled trial at a single center in China randomized infants post-HPE to seven or 14 days of intravenous antibiotics (cefoperazone/sulbactam and ornidazole) prior to an oral antibiotic regimen for 6 months (alternating sulfamethoxazole and cefaclor every two weeks) (33). There was no difference in the primary outcome of cholangitis occurrence at 6 months between the short- and long-term groups (62% vs. 70%, *P *= 0.27), but there was decreased early onset cholangitis (within one month) in the long-term group (61% vs. 38%, *P = *0.02). Older retrospective cohort studies have evaluated patients receiving antibiotic prophylaxis versus historical controls that did not receive antibiotics, with only Lally et al. (antibiotic *n* = 34, no antibiotic *n* = 7) reporting significantly decreased cholangitis incidence, while Wu et al. (antibiotic *n* = 16, no antibiotic *n* = 21) and de Vries et al. (antibiotic *n* = 124, no antibiotic *n* = 80) found no difference in cholangitis incidence (34–36).

### Ursodeoxycholic acid

Ursodeoxycholic acid (UDCA), a hydrophilic secondary bile acid, is used for a variety of cholestatic liver diseases (37). UDCA decreases progression to liver transplant and death in primary biliary cholangitis, and improves liver biochemistry (but not histologic progression, need for transplant, or mortality) in primary sclerosing cholangitis (38, 39). At very high doses (28–30 mg/kg/day) in an adult primary sclerosing cholangitis population, there is a doubling of severe adverse effects as well as an increase in a composite outcome of death, transplantation, or minimal listing criteria (40). This toxicity has been attributed to UDCA metabolism into lithocholic acid, which in animal models may lead to an obstructive cholangiopathy and bile duct destruction (41). UCDA has a choleretic effect through increased chloride and bicarbonate secretion, posttranscriptional modification of hepatobiliary protein levels of key ATP-binding cassette transporters, and cholangiocyte/hepatocyte protection (42–44). *In vitro* experiments also demonstrate that UDCA moderates the inflammatory response of peripheral blood mononuclear cells and human B cell lymphoma cells with decreased immunoglobulin and cytokine production (45).

Off-label administration of UDCA post-HPE is routine at many centers around the world, with surveys of European and Japanese centers finding near-universal use (31, 46). Despite this, a lack of high-quality literature exists to support a benefit or harm of UDCA. In a prospective single institution study of 16 children in France, after UDCA was discontinued at 18 months post-HPE, liver enzymes worsened in 13 children (47). Six months following resumption of UDCA, liver enzymes, bilirubin, and bile acids decreased, providing the most direct evidence for use of UDCA in biliary atresia. A retrospective cohort study at a single Egyptian hospital compared infants post-HPE who received UDCA (*n* = 108) versus those who did not (*n* = 33), suggesting a worse outcome in patients who received UDCA (48). A “successful” outcome, defined as absence of jaundice with aminotransferase levels below twice the upper limit of normal, was achieved in 24.2% that did not receive UDCA versus 10.2% of infants who did (*P *= 0.04), but there was no difference in the proportion that “failed” (75.7% vs. 77.8%, *P *= 0.49). The study was limited by treatment selection bias, unclear temporality of the UDCA initiation, and overall poor outcomes. The progression to transplantation as well as actual biochemical values were not reported. Given the paucity of investigation of UDCA in this population, well-designed multicenter randomized controlled trials are needed to both better understand the possible benefit or harm of this medication as well as determine the optimal dose.

### Nutrition

Children with biliary atresia are at high risk for malnutrition, growth failure, and vitamin deficiencies (especially fat-soluble vitamins) due to malabsorption, anorexia, and altered nutrient metabolism (49). Growth failure is associated with increased pre- and post-transplant mortality and graft failure (50, 51). Close surveillance combined with aggressive nutritional supplementation is critical. Initial recommendations include high protein intake (3–4 g/kg/day) and up to 130%–150% of recommended calories for age, with sequential stepwise escalation of nutritional support to nasogastric feeds and ultimately parenteral nutrition (49). Medium chain triglyceride supplementation is under study as a source of caloric dense, easily absorbable lipids (NCT05072626). Critically, medium chain triglycerides do not contain the essential polyunsaturated long-chain fatty acids necessary for growth and development. As growth failure is common in biliary atresia and often leads to early liver transplantation, further research is critical in this area to optimize nutrition, as well as further investigating detoxification genomics in biliary atresia (52).

### Antiviral therapy

Various viral infections have been proposed as possible inciting events in a subset of biliary atresia pathogenesis, with the greatest investigation of cytomegalovirus (CMV). Between 10% and 74% of biliary atresia patients have positive CMV serology depending on series, and CMV-positive patients have worse outcomes (53). Limited investigation has evaluated the role of antiviral therapy in this population. One retrospective single center trial found that in 36 CMV IgM+ patients, treatment with ganciclovir and/or valganciclovir in the early postoperative period until negative CMV serology resulted in greatly improved clearance of jaundice (75 vs. 21%, *P = *0.04) (54). Prospective investigation is critical to demonstrate efficacy of targeted antiviral therapy.

### Cholestyramine and phenobarbital

Cholestyramine and phenobarbital are used at some centers despite minimal evidence of effectiveness (31). Cholestyramine is a bile acid sequestrant, while phenobarbital is a choleretic that, in rats, increases the bile acid-independent portion of bile flow (55). The primary trial investigating these agents (from 1986) randomized infants with biliary atresia following surgical repair (primarily Kasai HPE) to no choleretic (*n* = 12), phenobarbital (*n* = 21), or cholestyramine (*n* = 23) for three months (56). There was no difference in total bilirubin or bile acid levels at three months in any group.

## Emerging therapies for biliary atresia

There are several emerging therapies under investigation for the treatment of patients with biliary atresia. Ongoing late-stage clinical trials of repurposed drugs that are FDA-approved for non-biliary atresia indications are shown in Table 1.

**Table 1:** Selected ongoing clinical trials investigating repurposed therapeutics for biliary atresia.

Drug	Identifier (s)	Phase	Design	Locations	Target Size	Status
Obeticholic Acid	NCT05321524	Phase II	Non-randomized, sequential assignment, open-label	>10 countries	32	Not Yet Recruiting
Odevixibat	NCT04336722	Phase III	Randomized, double-blind, placebo-controlled	>10 countries	200	Recruiting
Bezafibrate	JPRN-jRCTs031210066	Phase II	Non-randomized, single-arm, open-label	Japan (single site)	10	Recruiting
Maralixibat	NCT04524390; EUCTR2020-000974-22-GB	Phase II	Randomized, double-blind, placebo-controlled	USA, France, Canada, Poland, Germany, Italy, United Kingdom	72	Recruiting
Rituximab	ChiCTR2000031738	Phase IV	Non-randomized, controlled	China (single site)	6	Recruiting
Granulocyte-colony stimulating factor	NCT04373941	Phase II	Randomized, controlled, open-label	Pakistan, Vietnam	200	Recruiting
Mitomycin C	CTRI/2018/12/016495	Phase II	Randomized, controlled, open-label	India	50	Not Yet Recruiting

### Ileal bile acid transporter inhibitors

Odevixibat is an oral small molecule ileal bile acid transporter inhibitor FDA-approved for pruritus progressive familial intrahepatic cholestasis (PFIC). Inhibition of the ileal bile acid transporter prevents bile acid absorption in the terminal ileum, increasing fecal bile acid excretion. Importantly, this may lead to deficiency in fat-soluble vitamin absorption and the impact on growth and development is unknown. In the PEDFIC 1 trial, daily administration of odevixibat reduced serum bile acids and pruritis in children compared to placebo, and was well tolerated (57). Notably, the PEDFIC 1 trial only included type I and type II PFIC, which are distinct from type III and IV PFIC due to their low-GGT cholestasis. Odevixibat is currently under study in a randomized, double-blind, placebo-controlled phase III trial for biliary atresia (NCT04336722).

Maralixibat (Mirum Pharmaceuticals) is a second oral small molecule ileal bile acid transporter inhibitor FDA-approved for treatment of cholestatic pruritis in Alagille Syndrome (58). In the phase IIb randomized placebo-controlled ICONIC trial, maralixibat treatment reduced serum bile acids and pruritis in children with Alagille syndrome complicated by cholestasis and intractable pruritis (59). A multinational, randomized, double-blind, placebo-controlled phase II trial is currently recruiting patients with biliary atresia to evaluate maralixibat (NCT04524390).

### Obeticholic acid

Obeticholic acid is a semi-synthetic bile acid that is a potent Farsenoid X receptor (FXR) agonist FDA-approved to treat UDCA-resistant primary biliary cholangitis (60). FXR is a key ligand-modulated nuclear regulator of hepatic lipogenesis, insulin resistance, bile metabolism, and inflammation (61). In a phase III trial for UDCA-resistant primary biliary cholangitis, obeticholic acid decreased alkaline phosphatase compared to placebo, although pruritis was a common side effect (62). A phase I multicenter open-label single/multiple dose study investigating the safety and tolerability of obeticholic acid in biliary atresia patients is underway in Europe (NCT05321524).

### N-acetylcysteine

N-acetylcysteine is a hepatoprotective and antioxidant agent used for acetaminophen overdose that drives glutathione synthesis and increases bile flow (63, 64). Previous investigations of N-acetylcysteine in pediatric diseases such as parenteral nutrition-associated cholestasis and bronchopulmonary dysplasia demonstrated excellent safety, although it did not demonstrate efficacy (65–67). A phase II open-label single center trial (NCT03499249) is underway in the United States to evaluate if seven days intravenous infusion of N-acetylcysteine following HPE improves bile flow (68).

### Intravenous immunoglobulin

Intravenous immunoglobulin (IVIG) is a preparation of immunoglobulin G purified from donor plasma (69). Gamma globulins were first used for primary humoral immunodeficiency; IVIG was later introduced for autoimmune disease in idiopathic thrombocytopenic purpura (70, 71). The immunomodulatory and anti-inflammatory actions of IVIG involve both innate and adaptive immune cells, with effects extending beyond the half-life due to poorly understood mechanisms (72). Due to the proposed centrality of autoimmunity and inflammation in the pathogenesis of biliary atresia, IVIG has been investigated as a potential therapy.

The PRIME trial was a multicenter phase I/IIa trial performed at eight North American centers. Twenty-nine patients with biliary atresia received IVIG and were compared to a historical “placebo” control cohort (*n* = 64) from the START trial (73). Subjects received three 1 g/kg infusions of IVIG at days 3–5, 30, and 60 post-HPE. The primary outcome was safety: 89.7% of IVIG patients had a serious adverse event compared to 75% in the historical cohort, although no events were directly attributable to IVIG infusion. There was no difference in serum bilirubin, but there was a non-significant trend towards decreased transplant-free survival at 360 days in the IVIG group (58.6% vs. 70.5%, *P* > 0.05). Randomized controlled trials comparing IVIG and glucocorticoids are necessary.

### Other investigational therapies

Several small trials are underway to investigate other repurposed therapies for biliary atresia. Granulocyte-colony stimulating factor (GCSF) stimulates hematopoietic stem cell (HSC) and neutrophil production and mobilization. Typically used in myelosuppression, it recently has been investigated in various liver diseases due to the potential for HSCs to modulate hepatic inflammation and promote regeneration (74). GCSF is currently being investigated in post-HPE type 3 biliary atresia (NCT04373941). Autologous bone marrow mononuclear cell infusion has also been investigated in a small phase I/IIa open-label trial in Vietnam, although the primary outcomes assessed were safety-related and efficacy cannot be determined (75).

Rituximab is an anti-CD20 human/murine chimeric monoclonal antibody that targets B cells. Initially developed for B cell lymphoma, it under investigation for several inflammatory liver diseases. A small non-randomized clinical trial is currently enrolling patients in China (ChiCTR2000031738).

Bezafibrate is a peroxisome proliferator-activated receptor agonist that was found in a randomized, double-blind, placebo-controlled trial to decrease cholestatic pruritus in a combined population of primary sclerosing cholangitis and primary biliary cholangitis patients (76). A study at a single center in Japan for biliary atresia is underway (jRCTs031210066).

The installation of mitomycin C at the portal plate at time of HPE is being investigated in a randomized phase II trial due to its antifibrotic properties (CTRI/2018/12/016495).

The gut microbiome is altered in children with biliary atresia (77). Two small trials have investigated *Lactobacillus casei rhamnosus* (LGG) supplementation. A small randomized controlled trial found no difference in jaundice, cholangitis, or need for transplantation at two years between LGG (*n* = 14) and placebo (*n* = 16) (78). A second trial randomized patients to LGG (*n* = 10) or neomycin (*n* = 10) for cholangitis prophylaxis, finding a similar frequency of cholangitis episodes (although less than a small historical control with no prophylaxis) (79). Further research is necessary to evaluate the efficacy of probiotics for cholangitis prophylaxis and other outcomes.

## Discussion

Current adjuvant therapy for biliary atresia post-HPE varies depending on center and is often based upon expert opinion, with few high-quality randomized controlled trials to support individual interventions. Given the rarity of biliary atresia and variation in management, studies are often both underpowered and not necessarily generalizable. The development of multicenter research networks with the infrastructure to conduct rigorous investigations is an encouraging trend**.**

The challenge in finding novel therapies for biliary atresia is compounded by a poor understanding of its underlying pathogenesis and a lack of representative animal models (80). Investigating emerging therapies found to be beneficial in other cholestatic and inflammatory liver diseases is perhaps the most promising approach to improve the outcomes of post-HPE biliary atresia.

A substantial need exists for well-designed trials to both optimize current therapies and investigate candidate therapeutics for post-HPE biliary atresia. Until then, the single most effective evidence-supported intervention to improve native liver survival is younger age at time of HPE, highlighting the importance of screening and early diagnosis.
